# A Method of Hepatocyte Extraction Conjugated with HPLC is Established for Screening Potential Active Components inChinese Medicines—Probing *Herba Artemisiae Scopariae* as an Exemplifying Approach

**DOI:** 10.3390/molecules17021468

**Published:** 2012-02-06

**Authors:** Min Hong, Hong-Yu Ma, Xiang-Rui Wu, Yong-Qing Hua, Quan Zhu, Hong-Wei Fan

**Affiliations:** 1 National Standard Laboratory of Pharmacology for Chinese Medicine, Nanjing University of Chinese Medicine, Nanjing 210029, China; Email: blues-xr@163.com (X.-R.W.); qzhu40@yahoo.com.cn (Q.Z.); 2 Jiangsu Key Laboratory for TCM Formulae Research, Nanjing 210046, China; Email: huayongqing@126.com; 3 School of Chinese Pharmacy, Shengyang Pharmaceutical University, Shenyang 110016, China; Email: umhyu@yahoo.com.cn; 4 Department of Clinical Pharmacology, Affiliated Nanjing First Hospital of Nanjing Medical University, Nanjing 210006, China

**Keywords:** hepatocyte extraction, HPLC, *Herba Artemisiae Scopariae*, bifendate, liver protection

## Abstract

In order to establish an effective and quick method for screening potential bioactive compounds in Traditional Chinese Medicines (TCMs), hepatocytes were employed for extracting either bifendate, a clinical medicine for liver diseases, or chemicals in *Herba Artemisiae Scopariae* (*A. Scopariae*), a commonly used traditional Chinese medicine for remedying liver diseases such as hepatitis induced by viruses, chemicals or alcohol. After hepatocyte extraction the compounds were analyzed by HPLC, therefore this method was referrred to as hepatocyte extraction conjugated with HPLC (HE-HPLC). In the first part of this study, HE-HPLC showed that bifendate was extracted by hepatocytes and detected by HPLC-DAD which indicated the feasibility of this method. Then in the second part of the study, the potential active components in the *A. scopariae* extract were studied using HE-HPLC. Six chemicals in the *A. scopariae* extract, which could bind to hepatocytes *in vitro*, were detected by HPLC-DAD and three were identified as 7-hydroxy-coumarin (7-OH-C), capillartemisin A and 7-methoxy-coumarin, respectively. *In vitro* assays showed that 7-OH-C protected HL-7702 hepatocytes from H_2_O_2_ injury. The results indicated that these compounds could be extracted by hepatocytes, could be detected by HPLC and more importantly were bioactive. It is suggested that HE-HPLC is a useful method for screening potent active components in Chinese medicines used to treat liver diseases.

**Abbreviations:** *A. scopariae*: *Herba Artemisiae Scopariae*; HE-HPLC: hepatocyte extraction conjugated with HPLC; FW: final washing eluate; DE: desorption eluate; RRT: relative retention time; RPA: relative peak area; 7-OH-C: 7-hydroxycoumarin.

## 1. Introduction

Traditional Chinese medicines (TCMs) are one of the major clinical medical treatments in China and have been demonstrated to be effective by their use in clinical practice for thousands of years. However, scientists are constantly attempting to determine which chemical(s) contribute to the effects of TCMs. Not knowing the effective compounds in TCMs is an obstruction not only in the quality control of crude drugs but also in illustrating their therapeutic principle which delays the understanding of the value of TCMs. A procedure often used to determine the active chemicals in TCMs is extraction followed by pharmacological screening. The extraction and purification of compounds from TCMs is time-consuming and aimless. The screening of bioactive compounds carried out in animal models are also laborious and inappropriate for the direct screening of bioactive components in TCMs. Modern pharmacological studies have shown that drug action is usually demonstrated by the drug combining with receptors, enzymes or channels on cell membranes. Therefore, the interaction of compounds with cells has been successfully used as a basis for the hypothesis of bioactive components in Chinese medicines [1,2,3,4]. In order to identify a quick and specific method to screen bioactive components in a complicated system of TCMs used to prevent liver injury, rat primary hepatocytes, bifendate and *A. scopariae* were used as examples to establish a method referred to as hepatocyte extraction conjugated with HPLC (HE-HPLC) in this study. Bifendate is a clinically effective medicine used to treat liver diseases and it’s bioactivity involves decreasing alanine aminotransferase (ALT) and aspartate aminotransferase (AST) secreted by hepatocytes in chronic hepatitis B [1] and attenuating hepatic steatosis [2]. It was initially identified and extracted from *Fructus Schisandrae* and was detected by HPLC-DAD [3]. *A. scopariae* is a commonly used traditional Chinese medicine (TCM), either used alone or in formulae, for treating liver diseases such as hepatitis induced by viruses, chemicals or alcohol [4,5,6] and has anti-apoptotic properties [7]. However, the biologically active components in *A. scopariae* are not completely known. It was reported that 6,7-dimethylesculetin was the active chemical, with activity which protected the liver from injury induced by CCl_4_[8], however, there are many more active chemicals contributing to the effect of *A. scopariae* which are as yet unidentified. In this study, bifendate and the potential active components in the *A. scopariae* extract were explored by HE-HPLC to establish the method and to identify an exemplifying approach. 

## 2. Results and Discussion

### 2.1. Detection of Bifendate after Hepatocyte Extraction

**Figure 1:**
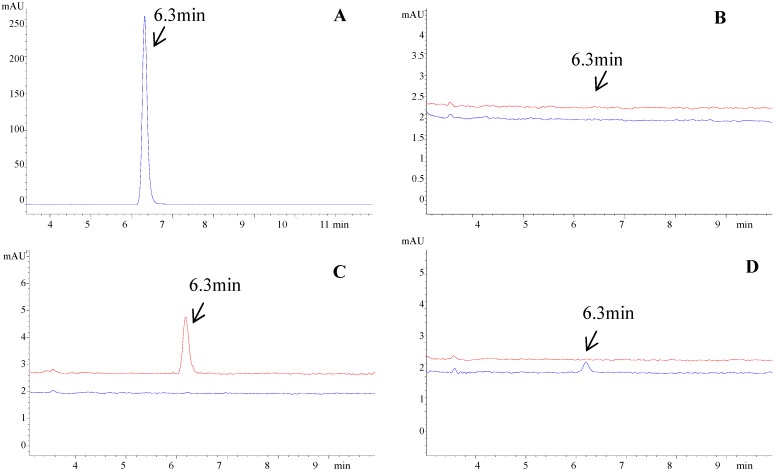
HPLC chromatogram of bifendate and detection of bifendate after extraction by hepatocytes. (**A**) Bifendate. (**B**) Final washing eluate (lower line) and desorption eluate (upper line) of the control group. There was no detectable bifendate at 6.3 min in the eluate. (**C**) Final washing eluate (lower line) and desorption eluate (upper line) of the medicated group. No detectable bifendate at 6.3 min was found in the final washing eluate. Bifendate was detected at 6.3 min in the desorption eluate which indicated that bifendate could bind to hepatocytes. (**D**) Washing eluate after four washes (lower line) and eight washes (upper line). Bifendate was detected after four washes while it was almost eliminated after eight washes.

Bifendate was detected at 6.3 min using the HPLC conditions described in subsection 2.5 (Figure 1A) After extraction by hepatocytes, bifendate was analyzed in the FW and DE in both the control and medicated groups. The results showed that there was no detectable peak in the FW and DE of the control group (Figure 1B). 

In contrast, after extraction by hepatocytes, bifendate was detected in the DE of the medicated group, but was not detected in the FW (Figure 1C) which indicated that bifendate could bind to hepatocytes and was detected by HPLC. Washing time was optimized by analyzing bifendate in different washing eluate. This showed that bifendate was still detected after four washes while it was almost eliminated after eight washes (Figure 1D). The effect of desorption time was also examined and showed no significant differences between 0.5 h, 1 h and 1.5 h (data not shown). Therefore, 0.5 h was selected for desorption in the study.

These results indicated that HE-HPLC may be effective for screening active components if they bind to cells. 

### 2.2. Identification of Potential Active Components in the *A. scopariae* Extract by HE-HPLC

#### 2.2.1. HPLC-DAD Analysis on the Fingerprint of *A. scopariae* Extract

##### 2.2.1.1. Selection of Suitable Chromatographic Conditions

**Figure 2:**
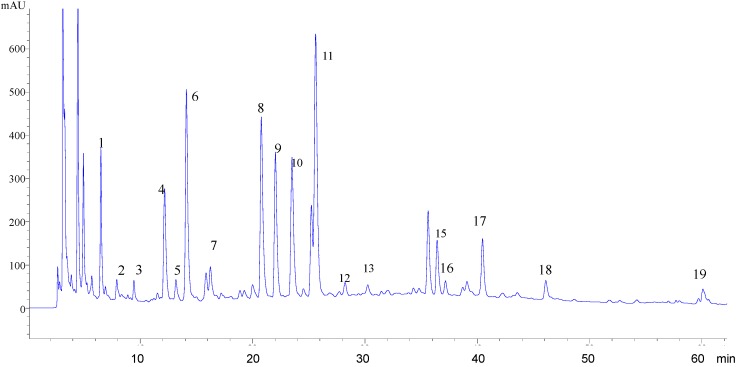
HPLC fingerprint of the *A. scopariae* extract. Nineteen “common peaks” were determined with respect to the common RRT and RPA in three batches of *A. scopariae* extract.

In the course of optimizing the separation conditions, the influence of the mobile phase was first investigated. In order to obtain the optimal elution conditions for the separation and determination of the constituents, different elution conditions using methanol-water and different concentrations of orthophosphoric acid in water were compared to obtain the most suitable mobile phase. The results showed that the best resolution and shortest analysis time was achieved when the methanol-water/orthophosphoric acid (100:0.05, v/v) system was used and by optimal gradient elution, all of the main peaks were well separated (Figure 2).

Selection of the detection wavelength was one of the key factors contributing to the reliable and reproducible HPLC fingerprints. The DAD detector was applied to select the optimum wavelength. This was observed at 280 nm where most of the main compounds in the chromatogram possessed strong UV absorbance, the number of peaks was increased and the signal was more sensitive compared with other wavelengths. Hence, 280 nm was selected as the detection wavelength for HPLC fingerprint determination of the *A. scopariae* extract.

##### 2.2.1.2. Method Validation

HPLC fingerprint determination is usually different from general assay methods. The authentication and identification of a drug and its products can be performed accurately using the chromatographic fingerprint, even if batches or concentrations vary among samples. Considering these characteristics of fingerprints, the relative retention time (RRT) and the relative peak area (RPA) of 19 “common peaks” were used to evaluate the quality of the samples. The method showed satisfactory linearity of 7-methoxycoumarin (R^2^ = 0.9976) in a linear range from 4.29 ~ 42.9 µg/mL. The limit of detection (LOD) of 7-methoxycoumarin was 3 µg/mL. The injection precision was determined by six replicate injections of the same sample in one day. The relative standard deviations (RSDs) of RRT and RPA were lower than 1.23% and 3.02%, respectively. The repeatability was assessed by analyzing six independently prepared samples of the *A. scopariae* extract. The RSDs of RRT and RPA were lower than 1.46% and 2.98%, respectively. The sample stability test was assessed by successive injections of the same sample at 0 h, 3 h, 6 h, 9 h, 12 h and 24 h. During this period, the solution was stored at room temperature. The RSDs of RRT and RPA were lower than 0.86% and 2.92%, respectively. The results indicated that the sample remained stable at 24 h. The results of injection precision, repeatability and the stability test indicated that this method was adequate, valid and applicable.

##### 2.2.1.3. Fingerprint of the *A. scopariae* Extract

The fingerprint of the *A. scopariae* extract was established by HPLC-DAD as a background with which binding components could be compared. Nineteen “common peaks” in the *A. scopariae* extract were determined, with respect to the common RRT and RPA in three batches of the *A. scopariae* extract (Figure 2). Peak number 17 was identified as 7-methoxycoumarin by comparison with the standard compound either by RRT or UV Spectra (figure not shown). The RRT and RPA of each peak were calculated by a comparison with 7-methoxycoumarin (Table 1). The fingerprint was performed to act as a reference for the detection of binding components.

**Table 1:** Relative tR and relative peak area of peaks in *A. capillaris* extraction.

Number of peaks	Relative retention time (RRT)	Relative peak area (RPA)	Percentage of total peak area (%)
1	0.160	1.496	5.35
2	0.195	0.440	1.57
3	0.233	0.399	1.42
4	0.300	1.671	5.98
5	0.325	0.422	1.51
6	0.348	2.480	8.87
7	0.401	1.371	4.90
8	0.514	2.403	8.59
9	0.544	1.879	6.72
10	0.581	2.042	7.30
11	0.633	4.968	17.76
12	0.697	0.356	1.27
13	0.747	0.482	1.72
14	0.880	1.334	4.77
15	0.900	0.834	2.98
16	0.918	0.517	1.85
17 *	1	1	3.58
18	1.139	0.445	1.59
19	1.486	0.399	1.43

* Peak number 17 was identified as 7-methoxycoumarin comparing with the standard compound (figure not shown). Relative retention time (RRT) and relative peak area (RPA) of each peak was calculated by comparison with 7-methoxycoumarin.

#### 2.2.2. Detection of the Components in *A. scopariae* after Extraction by Hepatocytes

**Figure 3:**
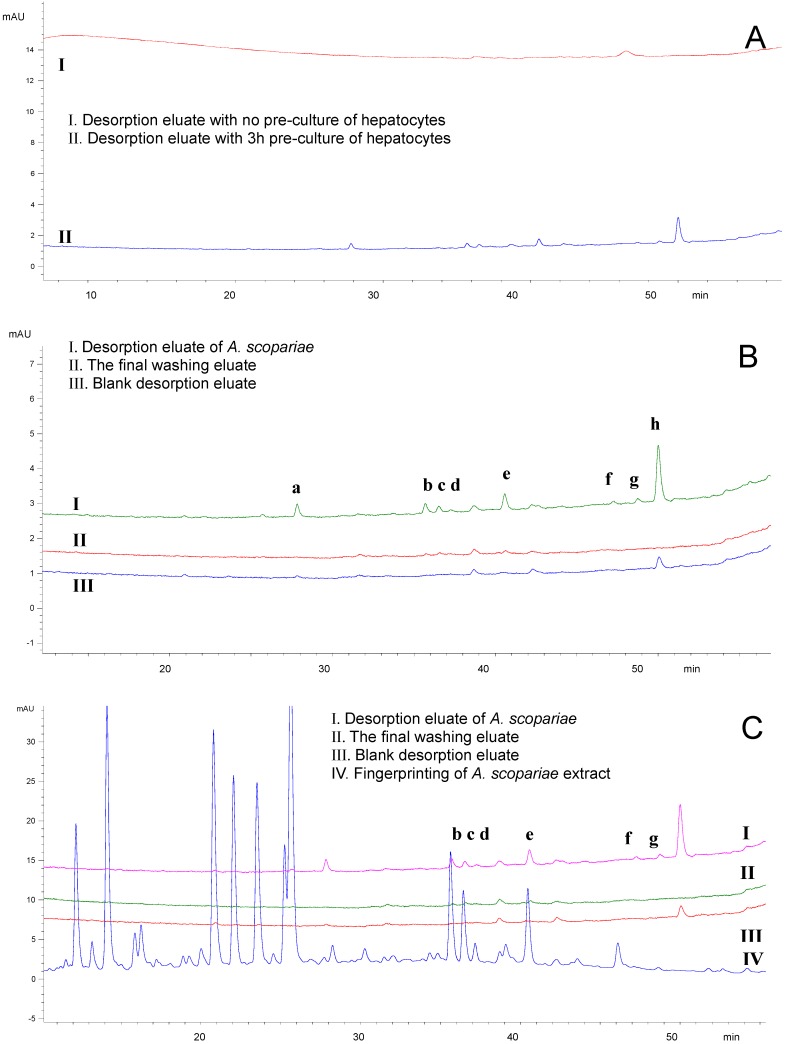
Detection of the components in *A. scopariae* after extraction by hepatocytes. (**A**) Effect of pre-culture of hepatocytes. No detectable components were found in the desorption eluate if the cells were not pre­cultured, while some peaks appeared if the cells were pre-cultured for 3 h. (**B**) Compared with the blank desorption and the final washing eluate, there were 7 peaks detected in the desorption eluate after treatment with *A. scopariae*.These peaks are marked “a” to “h”, respectively. (**C**) Components “b” to “g” were found in the fingerprint of *A. scopariae* extract.

The conditions employed were optimized in order to detect the components that bound to the hepatocytes. The results showed that pre-culture of hepatocytes before the extraction process is necessary. No detectable components were found in the DE if the cells were not pre-cultured, while some peaks appeared if the cells were pre-cultured for 3 or 6 h (Figure 3A). There were no marked differences considering the number of components between cells pre-cultured for 3 and 6 h (data not shown). Therefore, the hepatocytes were pre-cultured for 3 h before the extraction process. With regard to the concentration of the *A. scopariae* extract, desorption time and washing times, the conditions were optimized in the experiment to allow more components to be detected (data not shown). There were seven peaks detected in the DE after treatment with the *A. scopariae* extract and they were marked “a” to “h”, respectively (Figure 3B). Peak “h” was also detected in the control DE which indicated that this component was not found only in the DE treated with A. *scopariae*. After further comparison with the fingerprint of *A. scopariae* extract, peaks “b” to “g” were found in the fingerprint which indicated they were components from the *A. scopariae* extract (Figure 3C). As “a” and “h” were not found in the fingerprint this suggested that they might be components from the cells.

These results suggested that components “b” to “g” were compounds from A. *scopariae* which could bind to hepatocytes. To identify these components and evaluate their biological activities, compounds “b”, “c” and “e” (peak 14, 15 and 17 in the fingerprint) were extracted and purified for identification and bioactivity testing. Other components were not obtained at low contents in the crude herb which needs to be explored in the future. The three compounds obtained were confirmed to be peak “b”, “c” and “e” in the DE treated with *A. scopariae* extract by comparing RRT and UV spectra with that in the DE and the fingerprint. 

#### 2.2.3. Extraction and Identification of Compounds “b”, “c” and “e”

**Figure 4:**
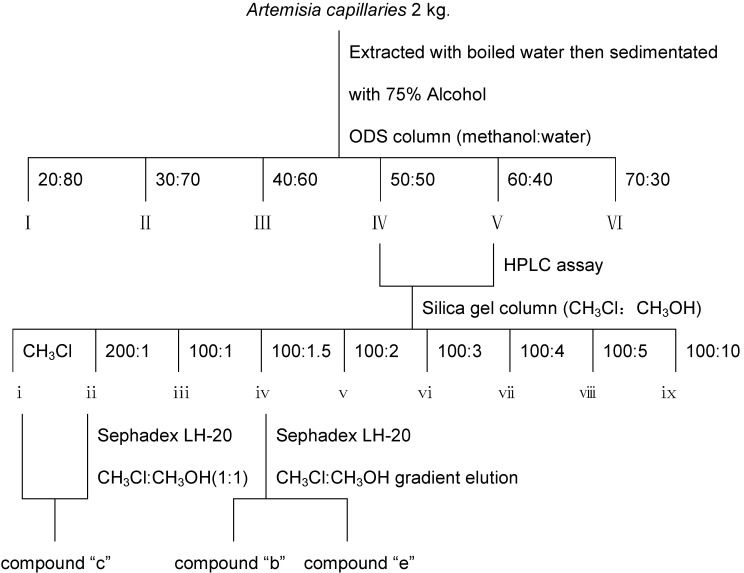
Extraction and purification of compounds extracted by HE-HPLC.

These compounds were obtained during the procedure described in Figure 4. Briefly, *A. scopariae*(2 kg) was extracted with boiled water then sedimented with 75% alcohol. The extract was separated by chromatography on an ODS column with a gradient eluted program of methanol-water. Of the six parts, parts IV and V were collected to undergo separation on a silica gel column with a gradient of chloroform-methanol (CH_3_Cl:CH_3_OH) and nine fractions were obtained. Fractions i and ii were pooled together and purified through Sephadex LH-20 with CH_3_Cl-CH_3_OH (1:1, v/v) to obtain compound “c”. Fraction iv was isolated by silica gel with a gradient eluted program of CH_3_Cl-CH_3_OH followed by Sephadex LH-20 with CH_3_Cl-CH_3_OH (1:1, v/v) to obtain “b” and “e”, respectively. Each step was analyzed by HPLC to track these compounds (Figure 4). 

#### 2.2.4. Extraction and Purification of Compounds Extracted by HE-HPLC

**Figure 5:**
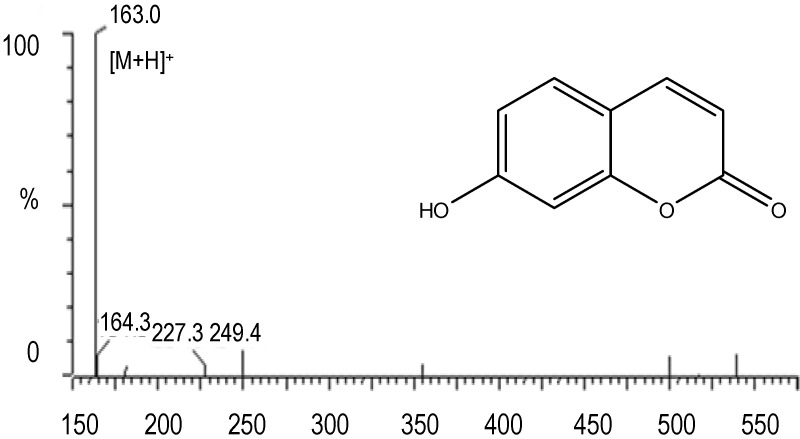
Mass spectra and structure of compound “b” which was identified as 7-hydroxycoumarin (7-OH-C).

Compound “b” was a white crystal. The MS spectrum of compound “b” is shown in Figure 5 and from a comparison of literature values it was observed that the mass spectrum of compound “b” was similar to the mass spectrum of 7-hydroxycoumarin (7-OH-C). 

**Figure 6:**
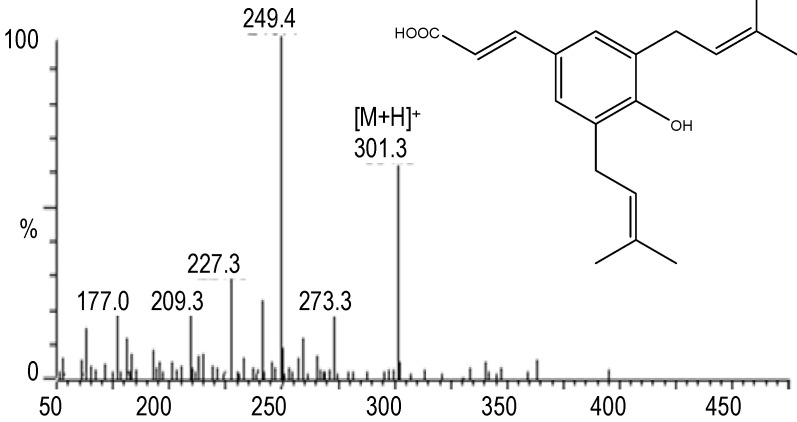
Mass spectra and structure of compound “c” which was identified as capillartemisin A.

The identification was further supported by ^1^H-NMR data: ^1^H-NMR (DMSO), δ: 7.87 (1H, d, *J* = 9.5 Hz), 7.42 (1H, d, *J* = 8.4 Hz), 6.69 (1H, dd, *J* = 8.4, 2.3 Hz), 6.60 (1H, d, *J* = 2.3 Hz), 6.05 (1H, d, *J* = 9.5 Hz). The structure of 7-OH-C is also shown in Figure 5. From the UV spectra, MS spectra (Figure 6) and a comparison with a previous report [9], compound “c” was identified as capillartemisin A. The structure is shown in Figure 6.

Compound “e” had the same Rt as peak 17 in the fingerprint of *A. scopariae* and was considered to be 7-methoxycoumarin. The identification was confirmed by IR spectrum and ^1^H-NMR data: IR (KBr): 1706, 1612, 1505, 1399, 1351, 1282, 1233, 1206, 1124, 1025, 980, 892, 829. ^1^H-NMR (CDCl_3_) δ: 7.66 (1H, d, *J* = 9.6 Hz), 6.27 (1H, d, *J* = 9.6 Hz), 7.40 (1H, d, *J* = 8.4 Hz), 6.87 (1H, dd, *J* = 8.4 Hz, 2.3 Hz), 6.84 (1H, d, *J* = 2.3 Hz), 3.90 (3H, S).

#### 2.2.5. Effect of Compounds on Proliferation/Viability of HL-7702 Hepatocytes

The effects of compound “b” and “c” on hepatocyte proliferation/viability were examined while compound “e” was not included because the quantity remaining was not sufficient for this assay. Five concentrations of compound “b” (0.1–1,000 μg/mL) and five concentrations of compound “c” (0.1–1,000 μg/mL) were added to HL-7702 hepatocytes for the bioactivity test. Each group included three replications. DMSO (0.1%) was added as a solvent control. A final concentration of 0.4 mM H_2_O_2_ was added to the cells (except the control group) 1 h later after treatment with the compounds. After 12 h of H_2_O_2_ injury, cell proliferation/viability was evaluated by the MTT assay and the cell proliferation/viability was represented by the A (absorbency) value. 

**Figure 7:**
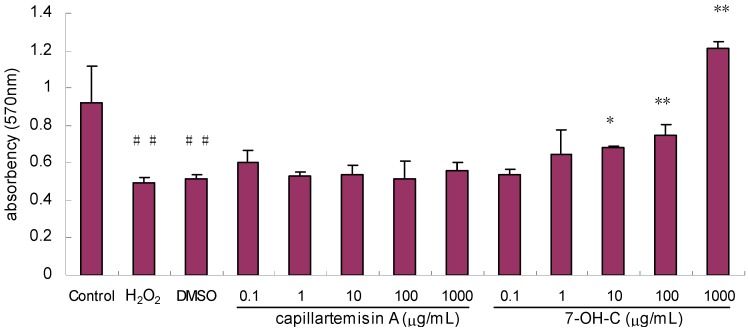
Effect of compounds on proliferation/viability of HL77-02 hepatocytes injured by H_2_O_2_. ^##^*p* < 0.01 *vs.* control; * *p* < 0.05, ** *p* < 0.01 *vs.* DMSO group.

High levels of H_2_O_2_ are observed during inflammatory and ischemic states of the liver and usually lead to cellular dysfunction and cytotoxicity [10]. In this study, H_2_O_2_ led to marked cell injury and compound “b” (7-OH-C) significantly protected hepatocytes from H_2_O_2_ injury at 10 μg/mL. It even demonstrated a noticeable trend in stimulating the proliferation of hepatocytes at high concentrations such as more than 100 μg/mL (Figure 7). Other circumstantial evidence that 7-OH-C interacted with hepatocytes is that 7-OH-C inhibited cell proliferation in a hepatoma-derived cell line (HepG2) in a concentration-dependent manner [11]. However, compound “c” (capillartemisin A) did not show this effect in this system and requires further study using other cell injury models. 

The results all above showed that (**1**) bifendate, a clinically effective compound used to treat liver diseases, was detected by HE-HPLC, (**2**) at least one compound extracted in *A. scopariae* by HE-HPLC could protect hepatocytes from injury to a certain extent *in vitro*. In another study on the active components in *Fructus Gardeniae* using this method, it was found that two characteristic compounds in the *Fructus Gardeniae* extract could bind to the hepatocytes, one of these compounds was geniposide [12] which can protect the liver from injury in mice [13] and possesses chemopreventive effects in early acute hepatic damage [14]. These results indicated that HE-HPLC was an effective method for screening potential bioactive compounds from complicated systems of TCMs.

Compared with traditional procedures for extracting and purifying as many compounds as possible from herbs followed by pharmacological screening, HE-HPLC does not need to purify the many compounds found in herbs but directly identifies target compounds in the complicated system which is more time efficient. Although the compounds extracted by HE-HPLC still need to be evaluated by bioassay, the number of screening compounds would be markedly reduced compared with the hundreds or more compounds in TCMs. Therefore, HE-HPLC may be a quick and effective technique for screening the active components in TCMs. Furthermore, the method could be extended to a wider field. In this study HPLC-DAD was used but some potential bioactive compounds might not be detected by HPLC-DAD. In addition, with other washing steps/conditions some compounds might be washed away or other compounds might have been bound to the cells. So this technique is subsensitive and some bioactive compounds might be missed. However, based on the method developed in this study, by using different extraction, different cells, different analyzing systems, such as fluorescence or LC/MS instead of DAD, and other binding/washing protocols, other or more active compounds may be found in TCMs. Nevertheless, like any *in vitro* assay, HE-HPLC has its limitations in that it can not identify those components acting with metabolites or acting on other related cells instead of on hepatocytes directly. 

## 3. Experimental

### 3.1. Materials and Chemicals

Bifendate was purchased from Xinchang Pharmaceutical Co., Ltd (Xinchang, Zhejiang, China). *A. scopariae* was purchased from the Ambulant Clinic of BaiCaoTang (an affiliated hospital of Nanjing University of Traditional Chinese Medicine). The mature plant of *A. scopariae*, “Yin Chen Hao” in Chinese, was utilized and the botanical origin of the materials was identified by Prof. Chun-gen Wang in Nanjing University of Chinese Medicine. A 7-methoxycoumarin standard was purchased from the National Institute for the Control of Pharmaceutical and Biological Products (Beijing, China). HL-7702 (L-02) a human hepatocyte cell line was obtained from Shanghai Cell Bank (Shanghai, China). Hydrogen peroxide (H_2_O_2_) was purchased from Shanghai Chemical Agent Limited Company (Shanghai, China). RPMI 1640 was a product of GIBCO (Grand Island, NY, USA). Fetal calf serum (FCS) was purchased from Minhai Biotechnology (Beijing, China). MTT and trypsin were purchased from Fluka (St. Quentin Fallavier, France) and AMRESCO (Cleveland, OH, USA), respectively. DMSO was from Sigma (St. Louis, MO, USA). Deionized water was prepared using a Millipore Milli Q plus system. Methanol was chromatographically pure. All other chemicals not mentioned here were of analytical grade from standard sources.

### 3.2. Preparation of Samples and Solutions

Bifendate: Bifendate granules (15 mg) were extracted with CHCl_3_ and methanol. The extract was then filtered and the solvent were evaporated. RPMI 1640 medium (150 mL) was added to DMSO (300 µL) containing bifendate (15 mg). The sample was obtained at a concentration of 1 mg/mL.

*A. scopariae* extract: *A. scopariae* was soaked in a 10 times volume of water and then decocted for 1.5 h three times. The filtered water extract was then slowly added to a quantitative volume of absolute ethanol to a concentration of 80% with stirring and the mixture was left overnight. The supernatant was concentrated and was filtered through a 0.45 μm filter membrane. The extract was ready for analysis and the concentration was 1.8 g crude drug/mL. 

D-Hanks (pH 7.4) was prepared with NaCl (8 g), KCl (0.4 g), KH_2_PO_4_ (0.06 g), Na_2_HPO_4_ (0.134 g), NaHCO_3_ (0.35 g), then metered volume to 1 L with deionized water. D-Hanks (100 mL) was used to adjust the pH to 4 with HCl.

### 3.3. Isolation and Culture of the Rat Primary Hepatocytes

Male SD rats were purchased from the Laboratory Animal Center of Nanjing Medical University and maintained in Nanjing University of Chinese Medicine under specific pathogen-free conditions. Rat primary hepatocytes were isolated using a procedure as described previously [15]. Briefly, the rats were anaesthetized and underwent liver perfusion with 0.5% collagenase type IV. The flow of perfusate was stopped as the cells digested thoroughly and filtered through double-deck absorbent gauze. The obtained hepatocytes were suspended in RPMI 1640 and washed three times by slow centrifugation (120 × g for 1 min). The isolated hepatocytes were cultured in RPMI 1640 containing 10% FCS and incubated at 37 °C under 5% CO_2_ for 3 h.

### 3.4. Method for Hepatocyte Extraction Conjugated with HPLC (HE-HPLC)

#### 3.4.1. Extracting Bifendate or the Components in *A. scopariae* Extract by Hepatocytes

The obtained rat primary hepatocytes were dispensed into two sets (control and medicated) with a density of 2 × 10^6^/mL. To the medicated set, either bifendate or *A. scopariae* extract was added at a concentration of 50 μg/mL or 90 μg/mL, respectively. To the control set, RPMI 1640 was added to the same volume. The two sets of cells were incubated at 37 °C under 5% CO_2_ for 1.5 h. The cultured cells were thoroughly washed 8 times with D-Hanks to remove the unbound components. The washing eluate was discarded with the exception of the last washing which was collected and used as one of the controls for HPLC analysis (final washing eluate, FW). Components bound to the cells were liberated by adding D-Hanks at pH 4 for 30 min followed by centrifuging at 1,000 rpm for 10 min. The supernatant with the released components was obtained after centrifugation and was referred to as the desorption eluate (DE). The obtained FWs and DEs were filtered through a filter membrane (0.45 μm) and then condensed using an SPE solid phase column extractor (B-type, C_18_, 500 mg). The solvate was obtained in the silica-bonded phase with six hold-up volumes of methanol. The cartridge was flushed with six to ten hold-up volumes of water. The sample was loaded and the eluate contained the unwanted components in water. The retained components were eluted with 1 mL of methanol and was filtered through the 0.45 μm membrane filter. The FWs and DEs were then ready for HPLC analysis.

#### 3.4.2. HPLC-DAD Analysis of Bifendate or the Components in the *A. scopariae* Extract

Analysis were performed on an Agilent 1100 series HPLC apparatus (Agilent Technologies, Palo Alto, CA, USA) equipped with a G1315GAD diode array detector, a G1311 quaternary gradient pump, an autosampler, a column-heater, a vacuum degasser and a DAD detector, connected to Agilent ChemStation software. The gradient hysteresis volume was 1.4 mL.

Conditions used for bifendate [3]: Agilent HC C_18_ column (4.6 mm × 250 mm, 5 μm) was used at 40 °C. The mobile phase was MeOH-Water (65:35, v/v). The flow rate was 1 mL/min and the sample injection volume was 10 μL. The wavelength of the DAD detector was 278 nm.

Conditions used for the *A. scopariae* extract: Fingerprint, washing eluate and desorption eluate of the *A. scopariae* extract were all analyzed under the following conditions: A HanBang Lichrospher C_18_ column (4.6 mm × 250 mm i.d, 5 μm) was employed at 30 °C. The flow-rate was 1 mL/min and the recording time was 65 min. The wavelength of the DAD detector was 280 nm. The binary gradient elution system consisted of methanol (A) and water (B) with 0.05% orthophosphoric acid. A + B = 100%. The gradient program was: 0 ~ 50 min, 10% ~ 60% A; 50 ~ 65 min, 60% ~ 95% A; 65 ~ 70 min, 95% ~ 10% A; 70 ~ 100 min, 10% A.

### 3.5. Mass Spectrometry

A Waters ZQ2000 liquid chromatography-mass spectrometer system (including a quaternary gradient pump, auto-sampler, column-incubator, equipped with an ESI ion source, 2695 liquid chromatography) was used and separation was achieved on an Agilent Zorbax C_18_ (5 μm × 250 mm × 4.6 mm, Agilent Co., Ltd.) at a temperature of 30 °C and a flow rate of 1.0 mL /min; Masslynx 4.0 High Chromatography workstation; The elution system was 0.1% formic acid water solution and methanol which included 0.1% formic acid with a proportion of 50:50 (v/v). The detection wavelengths of DAD were 210–400 nm. Post column split into mass detector, 0.4 mL/min.

The electrospray ionization source was performed in the positive and negative ionization mode in scan range 100–1,000. The MS operating conditions: Source Temperature, 110 °C; cone voltage, 40 V; Desolvation Gas Flow 290 L/h; capillary voltage, 2.9 KV; Desolvation Temperature 425 °C.

### 3.6. Evaluation of HL-7702 Hepatocyte Proliferation/Viability by MTT Assay

Hepatocyte injury was induced by hydrogen peroxide (H_2_O_2_) as described previously [10]. HL-7702 hepatocytes were cultured at 37 °C, 5% CO_2_ in an incubator with RPMI 1640 containing 10% FCS. Cells in the exponential phase of growth were added to a 96-well plate and cell injury was modeled by adding 0.4 mM H_2_O_2_. After 12 h of injury, MTT was added to the system and the supernatant was discarded after 4 h, then DMSO (150 μL) was added to each well. The plates were detected for absorbency (A value) at 570 nm after shaking for 10 min using a microplate spectrophotometer (Molecular Devices, Sunnyvale, CA, USA). The experiments were repeated three times.

### 3.7. Statistical Analysis

The data were expressed as means ± SD and analyzed by the Student’s *t*-test.

## 4. Conclusions

A HE-HPLC method was established in this study. Firstly bifendate, a clinically effective compound used to treat liver diseases, was analysed by this method and the results showed that bifendate could be detected. Then *Herba Artemisiae Scopariae* was analysed by HE-HPLC as an example of screening potential active components in Chinese medicines. The results showed that at least one compound extracted in *A. scopariae* by HE-HPLC could protect hepatocytes from injury to a certain extent *in vitro*. These indicated that HE-HPLC was an effective method for screening potential bioactive compounds from complicated systems of TCMs.

## References

[1] Cui S., Wang M., Fan G. (2002). Anti-HBV efficacy of bifendate in treatment of chronic hepatitis b, a primary study. Zhonghua Yi Xue Za Zhi.

[2] Pan S.Y., Yang R., Dong H., Yu Z.L., Ko K.M. (2006). Bifendate treatment attenuates hepatic steatosis in cholesterol/bile salt- and high-fat diet-induced hypercholesterolemia in mice. Eur. J. Pharmacol..

[3] Chen Z.P., Zhu J.B., Chen H.X., Xiao Y.Y. (2007). A simple hplc method for the determination of bifendate: Application to a pharmacokinetic study of bifendate liposome. J. Chromatogr. B Analyt. Technol. Biomed. Life Sci..

[4] Cai H., Song Y.H., Xia W.J., Jin M.W. (2006). Aqueous extract of yin-chen-hao decoction, a traditional chinese prescription, exerts protective effects on concanavalin a-induced hepatitis in mice through inhibition of nf-kappab. J. Pharm. Pharmacol..

[5] Chen F.P., Kung Y.Y., Chen Y.C., Jong M.S., Chen T.J., Chen F.J., Hwang S.J. (2008). Frequency and pattern of chinese herbal medicine prescriptions for chronic hepatitis in taiwan. J. Ethnopharmacol..

[6] Wang X., Lv H., Sun H., Liu L., Yang B., Sun W., Wang P., Zhou D., Zhao L., Dou S. (2008). Metabolic urinary profiling of alcohol hepatotoxicity and intervention effects of yin-chen-hao-tang in rats using ultra-performance liquid chromatography/electrospray ionization quadruple time-of-flight mass spectrometry. J. Pharm. Biomed. Anal..

[7] Lee T.Y., Chang H.H., Wu M.Y., Lin H.C. (2007). Yin-chen-hao-tang ameliorates obstruction-induced hepatic apoptosis in rats. J. Pharm. Pharmacol..

[8] Wang X.J., Li T.L., Sun H. (2004). Hepatoprotective effects of yin-chen-hao-tang and its constituent absorbed into blood after oral administration. Zhongguo Yao Li Xue Tong Bao.

[9] Okuno I., Uchida K., Nakamura M., Sakurawi K. (1988). Studies on choleretic constituents in artemisia capillaris thunb. Chem. Pharm. Bull.(Tokyo).

[10] Yazihan N., Ataoğlu H., Yener B., Aydin C. (2007). Erythropoietin attenuates hydrogen peroxide-induced damage of hepatocytes. Turk. J. Gastroenterol..

[11] Weber U.S., Steffen B., Siegers C.P. (1998). Antitumor-activities of coumarin, 7-hydroxy-coumarin and its glucuronide in several human tumor cell lines. Res. Commun. Mol. Pathol. Pharmacol..

[12] Hong M., Ma H.Y., Zhu Q. (2009). Establishment of hepatocyte extraction combined with hplc (he-hplc) and application in analysis of active components in the fruits of gardenia jasminoides extract. Zhongguo Zhong Yao Za Zhi.

[13] Peng J., Qian Z.Y., Liu T.Z., Rao S.Y., Qu B. (2003). Comparative studies on hepatic protective and choleretic effect of geniposide and crocetin. Zhongguo Xin Yao Za Zhi.

[14] Wang C.J., Wang S.W., Lin J.K. (1991). Suppressive effect of geniposide on the hepatotoxicity and hepatic DNA binding of aflatoxin b1 in rats. Cancer Lett..

[15] Seglen P.O. (1975). Protein degradation in isolated rat hepatocytes is inhibited by ammonia. Biochem. Biophys. Res. Commun..

